# Advances in Encapsulating Marine Bioactive Compounds Using Nanostructured Lipid Carriers (NLCs) and Solid Lipid Nanoparticles (SLNs) for Health Applications

**DOI:** 10.3390/pharmaceutics16121517

**Published:** 2024-11-25

**Authors:** Rita Favas, Hugo Almeida, Andreia F. Peixoto, Domingos Ferreira, Ana C. Silva

**Affiliations:** 1 UCIBIO (Applied Molecular Biosciences Unit), Laboratory of Pharmaceutical Technology, Faculty of Pharmacy, University of Porto, 4050-313 Porto, Portugal; 2 Associate Laboratory i4HB Institute for Health and Bioeconomy, Faculty of Pharmacy, University of Porto, 4050-313 Porto, Portugal; 3 Mesosystem Investigação & Investimentos by Spinpark, 4805-017 Guimarães, Portugal; 4 LAQV-REQUIMTE (Associated Laboratory for Green Chemistry of the Network of Chemistry and Technology), Departamento de Química e Bioquímica, Faculdade de Ciências, Universidade do Porto, 4169-007 Porto, Portugal; 5 FP-BHS (Biomedical and Health Sciences Research Unit), FP-I3ID (Instituto de Investigação, Inovação e Desenvolvimento), Faculty of Health Sciences, University Fernando Pessoa, 4249-004 Porto, Portugal

**Keywords:** healthcare, cosmetics, food supplements, marine bioactive compounds, lipid nanoparticles

## Abstract

As life expectancy rises and modern lifestyles improve, there is an increasing focus on health, disease prevention, and enhancing physical appearance. Consumers are more aware of the benefits of natural ingredients in healthcare products while also being mindful of sustainability challenges. Consequently, marine bioactive compounds have gained popularity as ingredients in cosmetics and food supplements due to their diverse beneficial properties. Nonetheless, the use of some of these compounds is restricted by their low stability and poor aqueous solubility, necessitating solutions to overcome these limitations. In this context, lipid nanoparticles, such as solid lipid nanoparticles (SLNs) and nanostructured lipid carriers (NLCs), have been investigated for their potential to protect and improve the absorption of molecules through various routes, including oral and cutaneous. Numerous studies have shown that nanoencapsulating these compounds and incorporating them into cosmetics and food supplements can be effective. However, this application remains unregulated at the global level and is not currently addressed by existing legislation. Additional in vivo studies in both animals and humans are necessary to fully assess safety concerns.

## 1. Introduction

As life expectancy rises and modern lifestyles continue to evolve, there is a growing focus on health, particularly in disease prevention and enhancing physical appearance. Consumers are increasingly recognizing the advantages of incorporating natural compounds into health products [1].

The marine ecosystem provides a rich source of active ingredients that can be incorporated into cosmetics and food supplements. Among marine organisms, algae, crustaceans, bacteria, and fungi stand out as promising sources of bioactive compounds with different properties, such as antimicrobial and antioxidant, which are promising at different stages of product development, from processing to formulation and storage [2]. This vast source of bioactive compounds includes fatty acids, carotenoids, and vitamins, each of which has a wide range of health benefits.

Fatty acids are essential components of fats and oils, playing a crucial role in regulating various physiological processes [3]. They are categorized as monounsaturated fatty acids (MUFAs) or polyunsaturated fatty acids (PUFAs) based on their length, degree of unsaturation, and the number of chemical bonds. PUFAs, particularly omega-3, are the predominant type of fatty acids found in the marine environment and can be sourced from algae (especially red macroalgae), marine by-products, yeasts, bacteria, and fungi, serving as alternative options to traditional seafood for human consumption [3,4].

Carotenoids are isoprenoid pigments in the yellow-to-red spectrum and play a crucial role in various physiological activities [5]. They are produced by all photosynthetic organisms and obtained through diet by other organisms who cannot synthesize them [6]. Carotenoids are categorized into two main groups based on their structure and chemical composition. The first group includes carotenes and hydrocarbons without oxygen, such as β-carotene and lycopene. The second group includes xanthophylls, which are oxygenated derivatives of carotenes, such as astaxanthin, fucoxanthin, and zeaxanthin [7].

Vitamins are essential for proper metabolism and have an impact on overall health. As the body cannot produce enough vitamins, humans must get them from food or supplements. Vitamins are classified based on their properties, whether they are water-soluble or fat-soluble. While fat-soluble (i.e., lipophilic) vitamins, such as A, D, E, and K, are easily stored upon absorption, water-soluble (i.e., hydrophilic) vitamins, such as C and B, are eliminated and not stored in the body [8,9]. Common sources of these vitamins include fish, algae, bacteria, fungi, corals, sea cucumbers, and sponges [10,11,12,13].

Various methods and carriers have been used in the cosmetic and food sectors, such as microparticles (i.e., microcapsules or microspheres) and nanocarriers (e.g., liposomes, nanoemulsions, and lipid nanoparticles) [14]. Due to the lipophilic nature of some bioactive compounds, they cannot be incorporated into aqueous formulations. Encapsulation techniques have, therefore, been explored to increase the solubility, stability, and activity of these compounds [15]. They are used in the food industry to preserve components from environmental conditions, mask unpleasant tastes, or extend product shelf life [16]. In cosmetics, they can improve skin hydration and epidermal delivery, increasing local compound concentration with consequent less/no systemic absorption [17]. Although microencapsulation has been extensively studied, nanoencapsulation techniques have been found to resolve some limitations, leading to the production of more stable carriers that improve the absorption of bioactive molecules [16]. Among these, lipid nanoparticles are gaining attention for their effectiveness in encapsulating, protecting, and promoting the absorption of lipophilic molecules [15,18,19]. Furthermore, hydrophilic bioactive compounds, such as polysaccharides and proteins, are being used to modify the surface of lipid nanoparticles. Polysaccharides and proteins are important macromolecules in various living organisms and are critical in promoting overall body function and balance. They serve as energy sources and are essential for molecular recognition, defense, tissue formation, energy storage, and cellular transport [20,21,22]. Polysaccharides derived from different marine sources can be categorized into marine animal polysaccharides (i.e., chitin and chitosan), marine plant polysaccharides (i.e., alginate), and marine microbial polysaccharides (i.e., glucan) [20,23,24]. Fish and algae are the major sources of marine proteins, with the most interesting and useful being collagen and gelatin. These polymers have been applied to increase the stability and compatibility of lipid nanoparticles in different products and processes [25].

This review aims to provide an overview of the State of the Art in the use of marine bioactive compounds encapsulated in lipid nanoparticles, namely, nanostructured lipid carriers (NLCs) and solid lipid nanoparticles (SLNs), in cosmetics and food supplements. Regulatory and safety aspects of the use of this type of nanoparticles and the future directions of this area of research are also discussed.

## 2. Lipid Nanoparticles

Lipid nanoparticles are spherical particles containing a solid matrix composed of one or more lipids, where the encapsulated molecules are solubilized or dispersed, which is stabilized by one or two emulsifying agents. Depending on their internal structure and composition, these nanoparticles can be classified as solid lipid nanoparticles (SLNs) or nanostructured lipid carriers (NLCs) [17,26,27].

**Figure 1:**
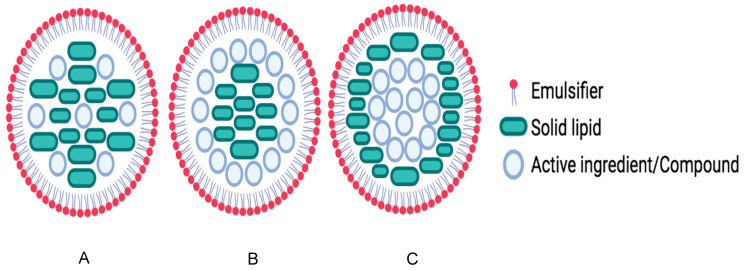
Different types of SLNs: (**A**) Homogenous matrix, (**B**) drug-enriched shell, and (**C**) drug-enriched core.

SLNs (Figure 1) were the first described and are composed of one solid lipid (5–40%) that contains dissolved or dispersed encapsulated molecules, one or two emulsifiers (0.5–5%), and water (q.s. 100%). The solid lipid forms a protective matrix that improves physical–chemical stability and slows the release of encapsulated molecules. The lipid matrix is solid at body temperature, protecting the molecules from degradation caused by environmental factors, such as light, heat, pH, and enzymes. Being composed of physiological lipids (known as Generally Recognized as Safe—GRAS), this type of lipid nanoparticle is biocompatible, exhibits good permeation through various body tissues, and can be easily incorporated in different pharmaceutical dosage forms for topical, oral, and parenteral administration [19,26,27]. Additionally, researchers have investigated the potential of targeting molecules encapsulated within surface-modified lipid nanoparticles for a range of therapeutic applications, such as neurological disorders, infections, and cancer. The findings so far have shown promising outcomes, highlighting the versatility and effectiveness of these nanoparticles in delivering treatments to specific sites within the body [28,29,30,31,32].

**Figure 2:**
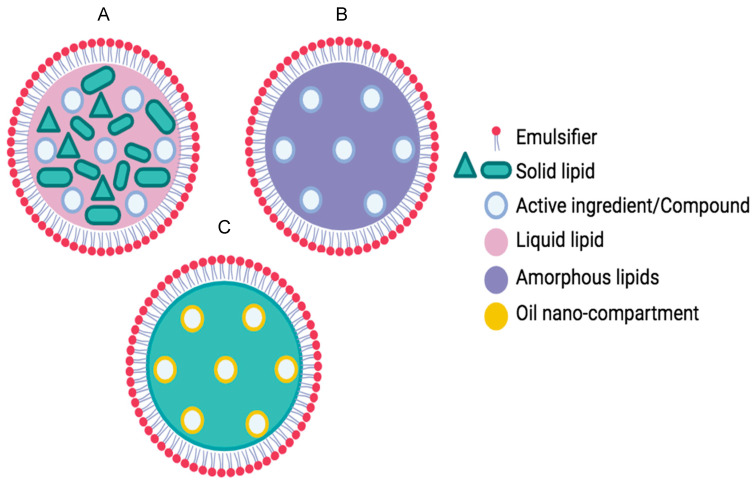
Different types of NLCs: (**A**) Imperfect, (**B**) amorphous, and (**C**) multiple.

Depending on the production method and the location of the encapsulated molecules, SLNs are classified as different types or models: homogenous matrix (Figure 1A), drug-enriched shell (Figure 1B), or drug-enriched core (Figure 1C). However, SLNs have limitations, mainly related to their low capacity to encapsulate molecules in the lipid matrix and excessive crystallization of the lipid during storage, leading to leakage of the encapsulated molecules. Thereby, more advanced nanocarriers derived from SLNs have been developed, nanostructured lipid carriers (NLCs), which have a solid matrix resulting from the combination of a solid lipid and a liquid lipid, the former in a higher percentage (Figure 2). Based on the location of the encapsulated molecules, there are different types of NLCs: Type 1, or the imperfect type (Figure 2A), occurs when the NLCs have an imperfect solid matrix structure and solid and liquid lipids are mixed in different nanostructures. The second type is the amorphous type (Figure 2B) and is obtained by mixing solid and liquid lipids in such a way as to prevent the formation of a solid crystalline structure, avoiding molecules from leakage during storage. The third type (Figure 2C) is the multiple type. Since lipophilic molecules are more soluble in liquid lipids than in solid lipids, in this type of NLC, there are oil moieties blended into the solid lipid, forming nano-compartments where the molecules are confined. Compared to SLNs, NLCs have been shown to have a higher loading capacity and a greater ability to prevent the release of molecules during storage by inhibiting or reducing the crystallization of lipids. These advantages have been attributed to the presence of the liquid lipid, which results in a more disorganized lipid matrix with more space to accommodate molecules [17,26,27,33,34,35].

Interested readers can consult the references provided for very comprehensive reviews of the characteristics of SLNs and NLCs.

### 2.1. Relevant Studies with Bioactive Marine Compounds Encapsulated in SLNs and NLCs

Given the various applications of marine bioactive compounds in healthcare and the possibility of encapsulating them in SLNs and NLCs to overcome their limitations, an analysis of published studies will provide good prospects for their potential use. Although most of the research focuses on the encapsulation of these compounds, some studies also present information on their potential health applications.

Additionally, marine bioactive compounds, such as chitosan, alginate, and gelatin, have been tested as coating polymers to modify the surface of SLNs and NLCs. They can increase their stability, performance, delivery efficiency, absorption rate, and biocompatibility [25]. The summary of key findings from relevant studies reveals a notable trend over the years. Starting from a single publication in 2006, there was sporadic research until a gradual increase beginning in 2017. The number of studies then surged, with the highest concentration occurring in 2020 and 2023, each with five published works. Table 1 provides details of the studies conducted between 2006 and 2023, with a total of 24 studies.

#### 2.1.1. Omega 3

Research on SLNs and NLCs loaded with omega-3 mostly focuses on their use as a component of the oil phase rather than as a bioactive compound. However, a few studies have explored the latest application. For instance, Serini et al. encapsulated α-linolenic and docosahexaenoic acid in SLNs. In the experiments, the researchers evaluated the physiochemical properties of SLNs and their effect on human colorectal cancer cells (HT-29 and HCT116 cell lines). Regarding docosahexaenoic acid and α-linolenic acid encapsulation, the particle size was 100 ± 1.8 nm and 842.2 ± 1.3 nm, the polydispersity index (PDI) was 0.220 ± 0.020 and 0.126 ± 0.017, and the encapsulation efficiency (EE) was 100% and 77%, respectively. The results show that after encapsulation, there was a significant increase in the uptake of α-linolenic acid (222.7%, *p* < 0.02) in cancer cells after 24 h of incubation. The incorporation of docosahexaenoic acid was also notably higher at 24 h when encapsulated in SLNs (277.2%, *p* < 0.009). At all tested concentrations, free docosahexaenoic acid and docosahexaenoic acid-loaded SLNs demonstrated a time-dependent inhibition of colorectal cancer cell growth. After 48 and 72 h, the 50 µM docosahexaenoic acid-loaded SLNs demonstrated a significantly greater inhibition of cell growth (68.6%, *p* < 0.01 and 80%, *p* < 0.001 in HT29 and HCT116 cells, respectively) compared to free docosahexaenoic acid at the same concentration (29.7% in HT-29 and 55.3% in HCT116). In addition, α-linolenic acid exhibited a significantly inhibitory effect on the growth of HT-29 and HCT116 cells after 48 h, with a more pronounced effect observed after 72 h. Notably, in HT-29 cells, α-linolenic acid-loaded SLNs were significantly more effective (*p* < 0.02) in inhibiting tumor cell growth than free α-linolenic acid at all the concentrations tested (5 µM, 10.7% vs. 34.6%; 10 µM, 12% vs. 36.3%; 50 µM, 2.3% vs. 38.2%). In HCT116 cells, α-linolenic acid-loaded SLNs demonstrated significantly enhanced efficacy than free α-linolenic acid at concentrations of 10 and 50 µM (22.5% vs. 29%, *p* < 0.05 and 29% vs. 79.1%, *p* < 0.001, respectively) [36].

The potential of using docosahexaenoic acid encapsulated in NLCs for peri-implantitis treatment was studied. The docosahexaenoic acid-loaded NLC had a particle size of 163.7 ± 2.0 nm, PDI of 0.118 ± 0.01, zeta potential (ZP) of 40.1 ± 1.3 mV, and EE of 78.13% ± 1.85%. The release of docosahexaenoic acid from the NLC showed a gradual and steady pattern over 144 h. According to the 2,2-diphenyl-1-pyridylohydrazinyl (DPPH) assay in vitro studies, the free-radical scavenging rate of the docosahexaenoic acid-loaded NLC was significantly higher (0.57 ± 0.03) than that of pure docosahexaenoic acid (0.17 ± 0.003, *p* < 0.001). Moreover, the docosahexaenoic acid-loaded NLC exhibited a superior inhibitory effect on the expression of cellular inflammatory factors compared to the pure form. In vivo studies on rats also showed that the docosahexaenoic acid-loaded NLC group displayed the most effective suppression of gingival inflammation after a 2-week reagent treatment [37].

#### 2.1.2. β–Carotene

Maretti et al. evaluated the skin penetration of a β-carotene-loaded NLC in humans (*n* = 4) by measuring the concentration of β-carotene in the stratum corneum after application to the skin and using pure β-carotene as a control. The results show that pure β-carotene was mainly retained in the outer layers of the stratum corneum (45%). In contrast, the β-carotene-loaded NLC demonstrated improved penetration into deeper skin layers, with 34% of β-carotene detected. The characterization studies reveal that the β-carotene-loaded NLC was spherical and showed a particle size of 222.8 ± 87.3 nm, PDI of 0.666, ZP of −43.46 ± 1.74 mV, and EE of 23.96 ± 3.13% [38].

The application of β-carotene in the food industry was also investigated by Rohmah et al. The study aimed to evaluate the bioavailability and antioxidant activity of a β-carotene-loaded NLC. For the experiments, an in vitro gastrointestinal tract model was used to simulate the normal course of ingested food through the mouth, stomach, and intestines. The particle size of the β-carotene-loaded NLC was 166.3 ± 0.19 nm, the PDI was 0.35 ± 0.1, the ZP was −26.9 ± 0.17 mV, and the EE was 91.2 ± 0.15%. The results show that the NLC has a superior capacity to encapsulate β-carotene compared to the other tested systems, such as a β-carotene emulsion, a β-carotene–Tween 80 phosphate-buffered solution, and a β-carotene–phosphate-buffered solution (*p* < 0.05). After 4 h of incubation, the release of β-carotene in the small intestine was significantly higher for the β-carotene-loaded NLC (233 μg/mL) compared to the other formulations, indicating that the NLC effectively released the β-carotene in the small intestine, reducing stomach degradation. Furthermore, the bioavailability of the β-carotene-loaded NLC (60.7%) in the small intestine exceeded the ones obtained from the emulsion (34.1%), the β-carotene–Tween 80 solution (23.4%), and the β-carotene–phosphate-buffered solution (8.7%). In terms of antioxidant activity, the β-carotene-loaded NLC exhibited moderate to strong antioxidant properties, with 2,2′-azino-bis(3-ethylbenzothiazoline-6-sulfonic acid) (ABTS) and DPPH assay values of 91.47 ± 1.9 and 24.72 ± 0.38%, respectively. Additionally, the IC_50_ of the β-carotene-loaded NLC was 7.0 μg/mL, while the pure β-carotene solution was 10.0 μg/mL, indicating enhanced antioxidant activity for the encapsulated β-carotene. In this study, the radical scavenging activity during the digestive process was also evaluated, which consistently demonstrated that the β-carotene-loaded NLC exhibited higher radical scavenging activity compared to the other tested formulations. These results suggest that NLCs may be a promising vehicle for application in functional food and beverages [39].

#### 2.1.3. Astaxanthin

Geng et al. optimized and assessed the stability, skin retention, and permeability of astaxanthin-loaded NLCs. The optimization process involved adjusting different independent variables, such as the solid lipid/liquid lipid ratio, total amount of lipids, astaxanthin concentration, and type and amount of emulsifiers, while the dependent variables were particle size, PDI, and EE. For the experiments, 21 formulations were prepared, and the optimized formulation showed stable nanoparticles with a spherical surface, a particle size of 67.4 ± 2.1 nm, a PDI of 0.26, and an EE of 94.3 ± 0.5%. This formulation was also non-irritating, homogeneous, and exhibited excellent stability. The in vitro release studies indicate that the cumulative release rate of astaxanthin from the NLC was 83.0 ± 3.4% for 48 h, while pure astaxanthin dissolved completely within 4 h. Moreover, the antioxidant and anti-linoleic lipid peroxidation activities of astaxanthin were effectively preserved following encapsulation in the NLC. The results of the skin penetration studies indicate that the cumulative permeability within 24 h was 174.10 ± 4.38 μg/cm^2^, and the retention was 18.60 ± 1.62 μg/cm^2^ for the astaxanthin-loaded NLC, while pure astaxanthin demonstrated permeability and retention of 295.20 ± 6.04 μg/cm^2^ and 8.00 ± 1.62 μg/cm^2^, respectively. Although the astaxanthin-loaded NLC showed a reduction in skin permeation, this formulation showed a sustained release profile compared with free astaxanthin, reducing the amount of free astaxanthin that can permeate through the skin. However, the skin retention of astaxanthin was effectively improved by encapsulation in the NLC, maximizing the local astaxanthin concentration in the skin while reducing its systemic absorption. These findings suggest that NLCs have the potential to serve as a carrier for astaxanthin, offering enhanced stability and improved skin retention capabilities [40]. Another study tested the ability of astaxanthin-loaded NLCs to reduce skin damage caused by radiotherapy. In the experiments, the effects of the astaxanthin-loaded NLCs on human fibroblasts and mice after exposure to X-irradiation were tested. The results show that the astaxanthin-loaded NLC had a particle size of 114.4 nm, ZP of −34.1 mV, and EE of 85.67%. Furthermore, it was observed that a concentration of 0.25 µg/mL of astaxanthin-loaded NLC successfully reduced reactive oxygen species (ROS) production by 81.6%, decreased DNA damage by 41.6%, and lowered cell death by 62.69% compared to the control. In the in vivo experiments, all six mice treated with an astaxanthin-loaded NLC were protected from acute skin damage after nine days of X-irradiation. In contrast, five out of six untreated mice exhibited grade 1 skin damage. Furthermore, after 28 days of treatment, the histological images indicate significant skin recovery with minimal differences in collagen fibers and sebaceous glands compared to normal skin [41]. Other studies have suggested that astaxanthin-loaded NLCs could be employed in the production of food supplements [42]. For example, researchers encapsulated astaxanthin in an NLC and analyzed its behavior during in vitro digestion. The particle size was 145.3 nm, the PDI was 0.468 ± 0.036, and the ZP was −30.8 ± 0.3 mV. The EE was 94.8 ± 1.0%, and a long-term stability study reveals that approximately 75% of astaxanthin remained encapsulated after 28 days of storage at 25 °C. In addition, the antioxidant activity test demonstrated that the astaxanthin retained its biological activity after encapsulation in the NLC (the DPPH assay was 95% for free astaxanthin and 100% for the astaxanthin-loaded NLC, *p* ≤ 0.05). The in vitro release study shows that free astaxanthin was rapidly released in the initial 6 h, reaching approximately 90% after 12 h, while encapsulated astaxanthin exhibited a more sustained release profile, with 88% released in 24 h. The results of the formulation’s digestion test indicate that the particle size and PDI remained stable during oral and stomach digestion. However, during intestine digestion, the particle size increased significantly (*p* < 0.05) to 227.6 ± 1.8 nm. This change was attributed to the presence of anionic components in the micelle mixture, such as bile salts, phospholipids, and free fatty acids. Additionally, the digestion process in the intestine initially occurred rapidly but then slowed down. The researchers concluded that astaxanthin was trapped in the inner oil phase and suggested that the lipid matrix might contribute to increased stability. These results suggest that NLCs can be a promising delivery system for astaxanthin, offering enhanced stability and a prolonged release effect [42].

Recent studies have suggested that encapsulating astaxanthin in NLCs or SLNs can enhance the treatment of neurodegenerative diseases via the nose-to-brain route. For instance, comparative in vitro experiments were carried out to evaluate the compatibility of an astaxanthin-loaded NLC and astaxanthin-loaded SLNs with nasal and neuronal cells. In this study, the antioxidant activity (neuroprotective effect) and the cellular uptake of astaxanthin-loaded SLNs and the astaxanthin-loaded NLC were also assessed. The results reveal that the astaxanthin-loaded SLNs had particle size of 106.967 ± 2.515 nm, PDI of 0.220 ± 0.017, ZP of −24.133 ± 0.379 mV, and EE of 99.99 ± 0.00%, while the astaxanthin-loaded NLC exhibited a particle size of 117.300 ± 2.163 nm, PDI of 0.222 ± 0.016, ZP of 23.267 ± 0.451 mV, and EE of 99.61 ± 0.04%. Additionally, both the astaxanthin-loaded NLC and astaxanthin-loaded SLNs were found to be safe for nasal and neuronal cells at concentrations up to 100 µg/mL. Regarding the neuroprotective effects, both formulations exhibited the ability to inhibit neurodegenerative pathways, such as oxidative stress. Notably, the astaxanthin-loaded NLC demonstrated superior neuroprotective effects against cytotoxicity induced by aggressors in comparison to the astaxanthin-loaded SLNs [43]. In addition, in the nose-to-brain route, a different study involving an astaxanthin-loaded NLC was carried out to test the in vivo effectiveness of this approach in slowing the progression of Alzheimer’s disease. The optimized astaxanthin-loaded NLC had a particle size of 142.8 ± 5.02 nm, PDI of 0.247 ± 0.016, ZP of −32.2 ± 7.88 mV, and EE of 94.1 ± 2.46% and remained stable at 4–8 ± 2 °C for six months. When administered to rats with Alzheimer’s disease-like symptoms, the astaxanthin-loaded NLC led to a significant decrease in oxidative stress, amyloidogenic pathway, neuroinflammation, and apoptosis while also showing improvement in cholinergic neurotransmission compared to a free astaxanthin solution [44].

#### 2.1.4. Lycopene, Fucoxanthin, and Zeaxanthin

Okonogi et al. developed a lycopene-loaded NLC for skin use, which had a particle size of 166 ± 4 nm, PDI of 0.15 ± 0.05, ZP of −74.6 ± 2.0 mV, and EE of 100 ± 0%. The in vitro release studies indicate a biphasic profile with faster release in the first 6 h, followed by sustained release over the following 18 h. The in vitro occlusion test reveals increased occlusive properties with higher lycopene-loading in the NLC. Additionally, the lycopene-loaded NLC exhibited higher antioxidant capacity (36.6 ± 0.4 mM/mg) compared to the placebo NLC (26.6 ± 0.1 mM/mg). The IC_50_ obtained by the DPPH assay was 14.1 ± 0.6 mg/mL for the lycopene-loaded NLC and 17.7 ± 0.4 mg/mL for the placebo NLC. Physical stability studies show that the particle size, PDI, and ZP of the lycopene-loaded NLC remained stable over 120 days when stored at different temperatures (4, 30, and 40 °C). Chemical stability data suggest that encapsulation in the NLC improved the stability of lycopene, particularly at lower temperatures (4 °C), which was demonstrated by the delay in lycopene degradation (0.4 μg/mL/day) [45].

Two distinct studies explored the potential use of fucoxanthin encapsulated in SLNs and NLCs for cosmetic applications. Cordenonsi et al. investigated the potential of a fucoxanthin-loaded NLC to prevent skin hyperproliferative diseases, particularly psoriasis. The fucoxanthin-loaded NLC had a particle size of 427.3 ± 5.7 nm, PDI of 0.309 ± 0.11, ZP of 21.21 ± 1.23 mV, and EE of 90.12 ± 2.51%. In vitro experiments on human fibroblast (NHDF) cultures show that up to a fucoxanthin concentration of 5 mM, there was no reduction in cell viability. Additionally, the fucoxanthin-loaded NLC decreased the expression of psoriatic markers by approximately 40%, suggesting their potential to manage skin hyperproliferation and inflammation [46]. In another study, Lee et al. assessed the efficacy of fucoxanthin-loaded SLNs to increase the effectiveness of sun protection. The results show that fucoxanthin-loaded SLNs had a particle size of 168 nm and PDI of 0.162. The sun protection factor (SPF) of the fucoxanthin-loaded SLNs was significantly higher (1.85 times) than that of the other formulations tested (three different chemical sunscreens), demonstrating a remarkable sun protection-enhancing effect [47].

The only study found on zeaxanthin involved the optimization of SLNs and an NLC loaded with the compound, which had the respective particle sizes of 179.16 ± 0.94 nm and 130.16 ± 1.58 nm; the PDI were 0.34 ± 0.01 and 0.30 ± 0.01; the ZP were −19.44 ± 1.19 mV and −21.49 ± 1.13 mV; and the EE were 81.14 ± 3.06% and 90.43 ± 2.85%. The researchers suggested that both SLNs and the NLC could have a wide range of applications, particularly as carriers for bioactive compounds in nutraceutical beverages [61].

#### 2.1.5. Vitamin A

The first documented studies on vitamin A-loaded SLNs date back to the early 2000s. Jenning et al. developed vitamin A-loaded SLNs and tested their efficacy in permeating porcine skin compared to a vitamin A nanoemulsion. The results reveal that SLNs significantly enhanced the stability of vitamin A compared to the nanoemulsion. The vitamin A-loaded SLNs had a particle size of 224 nm and a PDI of 0.205. Six hours after application, a substantial concentration of vitamin A (approximately 3400 ng) was detected in the stratum corneum and the upper epidermis. In contrast, the nanoemulsion only transported 2500 ng of vitamin A to the upper skin layers (*p* < 0.05). Surprisingly, after 24 h, a change in the distribution pattern was observed. The concentration of vitamin A in the upper epidermis decreased significantly to 900 ng (*p* < 0.05), while the concentration in the deeper skin layers increased from 0 to 250 ng (*p* < 0.05). In contrast, the distribution pattern of the vitamin A nanoemulsion did not change [48]. In another study, Pople et al. developed vitamin A-loaded SLNs with a particle size of 350 nm and compared their efficacy with the one from a vitamin A hydrogel. The in vitro release studies show that the vitamin A-loaded SLNs had a prolonged release of vitamin A for up to 24 h, with 54.38% of the compound released compared to 70% released from the vitamin A hydrogel. This extended release was attributed to the compound being embedded in the solid lipid core. In the in vitro penetration studies, after 24 h, the vitamin A concentration in the skin was almost 2 times higher with the vitamin A-loaded SLN formulation compared to the one obtained with the vitamin A hydrogel. Only 1.2% of the compound was detected in the receptor compartment of the vitamin A-loaded SLNs, while almost 10 times more was detected in the vitamin A hydrogel, resulting in a significantly higher amount of unabsorbed compound (around 72%) compared to the vitamin A-loaded SLNs (around 67%). The in vivo skin hydration studies in albino rats show that the application of the vitamin A hydrogel results in a slight change in the thickness of the stratum corneum, while the application of the vitamin A-loaded SLNs led to a substantial increase in its thickness: 3-fold compared to the hydrogel and 3.5-fold compared to the untreated skin. Additionally, the developed vitamin A-loaded SLNs showed a primary irritation index of 0.00, with no observed erythema or edema [49].

The potential use of vitamin A-loaded SLNs and vitamin A-loaded NLCs in the food industry has recently been investigated. The study focused on ensuring the stability and oral bioavailability of these nanoparticles under food processing conditions. Six formulations were developed for the experiments, from which two were selected for further investigation. The particle size, PDI, ZP, and EE for the vitamin A-loaded SLNs were 223 ± 10 nm, 0.171 ± 0.008, −25 ± 1 mV, and 97 ± 3%, respectively. For the vitamin A-loaded NLC, the corresponding values were 228 ± 7 nm, 0.146 ± 0.007, −22 ± 1 mV, and 90 ± 1%. The results of the stability tests show that the vitamin A-loaded SLNs and the vitamin A-loaded NLC remained stable for one month after storage at room temperature (25 °C). Furthermore, exposure to typical food processing temperatures (60 and 70 °C) did not significantly change the nanoparticle properties, although these temperatures were above the melting point of the solid lipid used in the nanoparticles (43 °C). The vitamin A-loaded SLNs and the vitamin A-loaded NLC were also tested in different media, including phosphate buffer at pH 5, highly concentrated sucrose solution, and high-ionic-strength sodium chloride solution. Both the vitamin A-loaded SLNs and vitamin A-loaded NLC showed stability in all the tested media, with variations of < 10% compared to fresh formulations not exposed to the various media. Afterward, to simulate gastric conditions, both tested formulations were subjected to a simulated gastric environment containing an acidic pH, salts, and gastric enzymes. Despite an increase of 0.03 in the PDI after two hours, the size of the vitamin A-loaded NLC remained approximately the same. A similar situation was observed for the vitamin A-loaded SLNs. Furthermore, after two hours in a simulated gastric environment, approximately 80% of the encapsulated vitamin A remained intact in the SLNs and NLC, suggesting their potential to deliver the compound to the small intestine where it can be absorbed [50].

#### 2.1.6. Vitamins D, E, and K

The potential of using NLCs to improve the effectiveness of vitamin D3 against breast cancer cells has been investigated. A vitamin D3-loaded NLC showed a particle size of 87 ± 5 nm, PDI of 0.24, and ZP of −12.2 ± 4.86 mV. The 3-(4,5-Dimethylthiazol-2-yl)-2,5-Diphenyltetrazolium Bromide (MTT) assay showed that the vitamin D3-loaded NLC was more successful in inhibiting cancer cell proliferation compared to a free vitamin D3 solution (*p* < 0.05), reducing cell proliferation from 49 ± 7.2% to 37 ± 5.1%, respectively. Furthermore, the vitamin D3-loaded NLC increased the percentage of cells in the apoptotic phase to 40 ± 3.45% (*p* < 0.05). These findings suggest that a vitamin D3-loaded NLC can improve the efficacy of chemotherapeutics in breast cancer patients [51]. Another study suggested using an oral vitamin D3-loaded NLC to improve the management of inflammatory bowel disease. The vitamin D3-loaded NLC had a particle size of 110 ± 4 nm, PDI of 0.23 ± 0.01, and ZP of −17.10 ± 0.30 mV. In the in vivo studies, when the vitamin D3-loaded NLC was orally administered to mice, the concentration of vitamin D3 in the colon was observed to be three times higher than the basal level, persisting for at least 12 h (*p* < 0.01). Subsequently, the researchers assessed the effects of the vitamin D3-loaded NLC on intestinal inflammation. The results demonstrate that although the symptoms worsened in mice treated with the free vitamin D3 solution and the vitamin D3-loaded NLC, the group tested with the vitamin D3-loaded NLC experienced progressive mitigation of clinical symptoms from day 3, and it suppressed a decrease in body weight from day 6. Additionally, the histological examination of colonic tissue showed significantly reduced inflammatory manifestations, immune cell infiltration, and crypt destruction in the group treated with the vitamin D3-loaded NLC (*p* < 0.01) [52].

Eiras et al. developed a vitamin E-loaded NLC and assessed its potential for skin application. The characterization of the NLC loaded with vitamin E showed that the particles had a consistent size in the nanometer range, with 90% having a size of less than 328 nm and 50% having a size of less than 105 nm, even after 7 months of storage. The results of in vitro studies in human keratinocytes (HaCAT) indicated that the vitamin E-loaded NLC is biocompatible (0.1–10 μg/mL) and non-irritant (with a score of 0.00), being suitable for skin application. From these findings, the researchers proposed using a vitamin E-loaded NLC in cosmetic and dermatological formulations to improve skin hydration [53]. Later, the same researchers conducted a more comprehensive study with this formulation, where they found no significant differences in particle size after incorporating a vitamin E-loaded NLC into a hydrogel (D90 = 386 ± 0.00 nm vs. D90 = 397 ± 0.021 nm). The biocompatibility studies were assessed by the MTT assay, where it was observed that the vitamin E-loaded NLC was more cytotoxic to human keratinocytes before incorporation in the hydrogel (IC_50_ = 14.38 μg/mL vs. IC_50_ = 28.74 μg/mL, *p* < 0.001). In vitro and in vivo tests reveal that the vitamin E-loaded NLC demonstrated superior occlusive properties (80% vs. 67%) and significantly reduced (12 g/H·m^2^) the skin’s transepidermal water loss (TEWL) compared to a vitamin E nanoemulsion (14 g/h·m^2^) (*p* < 0.05). In this study, the researchers demonstrated the safety and hydration potential of the hydrogel containing a vitamin E-loaded NLC for skin application. However, the need for more in vivo experiments over extended periods and involving more volunteers to confirm this evidence was emphasized [54]. In another study, the effectiveness of a vitamin E-loaded NLC was examined for its moisturizing and anti-aging properties. The optimized vitamin E-loaded NLC had a particle size of 82 nm, a PDI of 0.261, a ZP of −28.6 mV, and an EE of 95.83 ± 0.02%. The stability study reveals that the nanoparticles maintained their spherical shape and physical stability for 12 weeks after storage at room temperature. The release profile indicated that 30% of the vitamin E was released within the first 5 h, with 70% released after 24 h. The vitamin E-loaded NLC was incorporated into a hydrogel, and in vivo tests were performed on 13 human volunteers over 12 weeks to assess skin moisture content and mechanical properties. In the skin irritation test, all volunteers showed no signs of erythema after 48 h of exposure to the hydrogel containing the vitamin E-loaded NLC. However, 3 out of 13 volunteers experienced mild erythema when exposed to the vitamin E hydrogel alone. This suggests that the free form of vitamin E can be irritating to the skin, while the NLC provides a protective effect against this irritation. Additionally, throughout the 12-week study period, the hydrogel containing the vitamin E-loaded NLC enhanced the skin elasticity capacity (*p* = 0.0319) and consistently increased skin moisture levels by 80%, while a vitamin E hydrogel only resulted in a 20% increase in moisture levels (*p* < 0.01) [55]. In a different approach, the researchers evaluated the effectiveness of encapsulating vitamin E in SLNs to reduce the negative effects of anemia treatment. The characterization of the vitamin E-loaded SLNs revealed a particle size of 228.2 ± 3.5 nm, a PDI of 0.34 ± 0.02, a ZP of −8.92 ± 2.2 mV, and an EE of 99.9 ± 0.1%. The in vitro release studies reveal that approximately 65% of the encapsulated vitamin E was released after 24 h in simulated gastrointestinal media at 37 °C. Furthermore, the vitamin E-loaded SLNs demonstrated good hemocompatibility at various concentrations. After 3 h of incubation, there was minimal hemolysis (less than 0.3%). Even after 24 and 48 h, the level of hemolysis remained low, with less than 3.0% at the highest SLN concentration. It is important to note that biomaterials causing hemolysis of less than 5% are generally considered safe for human use, according to ISO/TR 7406. These results indicate their potential for clinical applications. Notably, the vitamin E-loaded SLNs significantly boosted lymphocyte cell proliferation by approximately 150% and decreased DNA damage caused by iron treatment [56].

Regarding vitamin K, only one significant study involving the encapsulation of vitamin K1 in SLN for oral delivery was found. In the experiments, seventeen different formulations were prepared and the most effective one was chosen for further tests. The particle size, PDI, ZP and EE for the vitamin K-loaded SLN were 132 nm, 0.17 ± 0.02, and −26.83 ± 2.83 mV, 98%, respectively. This formulation was shown to be stable in a 54-h in vitro release study in simulated gastric and intestinal media and after 4 months storage at 25 °C [57].

#### 2.1.7. Polymer Surface Coating

##### Chitosan

Almeida et al. aimed to evaluate the efficacy of chloroaluminium phthalocyanine-loaded NLCs coated with chitosan in the photodynamic therapy of skin cancer. A chloroaluminium phthalocyanine-loaded NLC coated with chitosan showed a particle size of 231.5 ± 5.8 nm, a PDI of 0.18 ± 0.01, a ZP of +19.96 ± 0.3 mV, and an EE of 96%. It is important to highlight that the chitosan coating resulted in a larger but equally viable NLC. Ex vivo studies show that the phthalocyanine-loaded NLC coated with chitosan significantly retained the compound in the skin after 2 h (5.8 ng) and 4 h (581 ng), with no detection in the bloodstream, indicating limited systemic exposure and no potential adverse events in patients. The biocompatibility test performed on L929 fibroblasts showed that the phthalocyanine-loaded NLC coated with chitosan did not induce cytotoxicity at any of the concentrations tested, indicating that the chitosan coating did not affect the biocompatibility of the NLC. It was also observed that the phthalocyanine-loaded NLC coated with chitosan was localized around the cellular nucleus. The photodynamic therapy tests performed on BF16-F10 melanoma cells showed that the phthalocyanine-loaded NLC coated with chitosan did not have toxic effects when not exposed to irradiation. However, after irradiation, there was a 50% reduction in cell viability (*p* < 0.001). These results highlight that the chitosan coating improved stability and biocompatibility and facilitated drug passage through the skin [58].

##### Alginate

Costa-Fernandez et al. developed an NLC co-encapsulated with vitamin E and quercetin and coated with alginate to investigate its potential to promote wound healing and evaluate the influence of the alginate coating in enhancing the penetration of the encapsulated compounds into the skin damaged by an incision. The results show that the alginate-coated NLC co-encapsulated with vitamin E and quercetin had a particle size of 321.2 ± 18.3 nm, a PDI of 0.23 ± 0.02, a ZP of −16.0 ± 3.0 mV, and an EE of 85% for quercetin and 92% for vitamin E. The irritation potential of the alginate-coated NLC was assessed by the Hen’s Egg Test–chorioallantoic membrane, showing it did not induce bleeding, lysis, or coagulation. Regarding the in vitro release, it was observed that after 16 h, 44.2% of quercetin and 32.3% of vitamin E were released. Furthermore, in ex vivo permeation studies, the experiments were performed on intact and damaged skin. In the intact skin, the permeation of the alginate-coated NLC co-encapsulated with vitamin E and quercetin was higher compared to a conventional formulation containing the same compounds. In the damaged skin, higher penetration of vitamin E and quercetin was observed with both formulations. The delivery of vitamin E to the epidermis and dermis increased by 1.9-fold and 2.3-fold for quercetin compared to the intact skin. The TEWL was also reduced (from 3 g/H·m^2^ to −7 g/h·m^2^) when using the alginate-coated NLC co-encapsulated with vitamin E and quercetin and water as the control. The results of this study highlight the advantages of using polysaccharides, such as alginate, as bioadhesive polymers to promote the penetration of compounds into the upper layers of skin [59].

##### Gelatin

Gelatin, a natural biopolymer (i.e., a protein composed of long chains of amino acids), is widely used in the food industry because of its advantages, such as non-toxicity, low cost, availability, biocompatibility, and biodegradability. Studies have shown that gelatin can stabilize nanocarriers due to its amine and carboxylic groups. Malekmohammadi et al. conducted a study aiming to synthesize a gelatin-coated NLC to encapsulate sage extract to inhibit microbial growth and lipid oxidation in beef burgers. The optimized sage extract-loaded NLC coated with gelatin had a particle size of 100.4 nm, PDI of 0.36, ZP of −18.4 mV, and EE of 80%. The results of the DPPH assay show that the sage extract-loaded NLC coated with gelatin exhibited significantly higher antioxidant activity than the free extract after 30 days of storage at 25 °C (*p* < 0.05). A higher inhibitory effect against *E. coli* compared to the free extract was also observed in the minimum inhibitory concentration (0.1 ± 0.00 vs. 0.2 ± 0.00 mg/mL) and the minimum bactericidal concentration (0.1 ± 0.00 vs. 0.3 ± 0.00 mg/mL) tests. Furthermore, incorporating the formulation into beef burgers increased their oxidation stability during 90 days of storage at 4 and −18 °C (*p* < 0.05). The sage extract-loaded NLC coated with gelatin also effectively decreased the total counts of various bacteria, yeasts, and molds in treated beef burger samples during storage in comparison to the controls (all *p* < 0.05). The sensory tests showed no significant differences in the color, odor, texture, and flavor attributes of the beef burger samples immediately after preparation. However, over time, the treated samples showed greater acceptability. These improved sensory properties were attributed to reduced proteolytic and lipolytic reactions, as well as reduced microbial activities. Overall, the study suggests that sage extract-loaded NLCs coated with gelatin can be an effective preservative for extending the shelf life of beef burgers [60].

## 3. Regulatory and Safety Concerns of Lipid Nanoparticles for Healthcare Applications

### 3.1. Cosmetics

Notwithstanding the regulatory frameworks governing the global market for nanocosmetics, the recognition of nanomaterials as cosmetic ingredients remains inconsistent across jurisdictions [62]. Consequently, each country adheres to its legal system. Given that the European Union (EU) and the United States of America (USA) represent the two most significant markets for cosmetics products, their respective regulatory frameworks are of significant importance. However, there are notable discrepancies between the two. Prior to 2023, the USA did not require cosmetic product registration, while the EU has mandated registration since 2013, especially for products containing nanoparticles. Furthermore, notification to the European Commission (EC), including information on the identification, specifications, toxicological profile, and safety of nanomaterials, is required. In cases of uncertainty regarding the safety of a nanomaterial, the EC can request an opinion from the Scientific Committee on Consumer Safety (SCCS) [63].

Under the stipulations set in the EC Regulation 1223/2009, the term “nanomaterial” is defined to regulate cosmetics products as a deliberately insoluble or bio-persistent manufactured material that has external dimensions (one or more) or an internal structure within the range of 1 to 100 nanometers [63]. On the other hand, in the USA, the Food and Drug Administration (FDA) oversees the use of nanomaterials in cosmetics through the Federal Food, Drug, and Cosmetic Act (FFDCA). However, the FDA does not have a legal definition for nanotechnology, does not approve the ingredients in cosmetic formulations, and does not require manufacturers to disclose the presence of nanomaterials in their products. The FDA has thus far refrained from making a definitive determination regarding the inherent safety or potential risks associated with nanotechnology [64]. As a consequence, the FDA has created the National Nanotechnology Initiative (NNI) and the Nanotechnology Task Force (NTF) intended to assess the restrictions required for nanotechnology products. These entities have published two documents addressing the safety issues of nanotechnology and cosmetics, which merely make recommendations. The first document is concerned with the determination of a material’s classification as a nanomaterial based on the size of its particles and their properties/phenomena. In particular, it considers whether the material or final product is (a) intentionally designed to have at least one external dimension, internal dimension, or surface structure within the nanoscale range (approximately 1 nm to 100 nm) and (b) meant to exhibit properties or phenomena, including physical, chemical, or biological effects, attributed to its size, even if these dimensions extend beyond the nanoscale range to one micrometer (1000 nm). It is important to highlight that the properties of nanomaterials relevant to safety, effectiveness, performance, quality evaluation, public health impact, and product regulatory status can be linked to materials with one or more dimensions larger than the 1–100 nm range. In a succeeding document, the FDA advises a thorough assessment of safety by characterizing the nanomaterial itself and examining a wide range of chemical and physical properties. This involves evaluating the toxicity, absorption, distribution, metabolism, and excretion of the particles [62,65].

The scientific community has been debating whether the use of insoluble nanoparticles in cosmetics constitutes a health risk. The results so far have been inconclusive due to inconsistent findings and a lack of long-term toxicological studies. Safety evidence originates mainly from the EU, which has strict regulations [65]. Regarding the use of SLNs and NLCs in cosmetics, these can be classified as nanomaterials according to the EU standards, as they consist of water-insoluble materials, such as solid and liquid lipids. However, these lipids are similar to the body’s natural lipids. They can readily adhere to and interact with the outer layer of the skin, causing lipid rearrangement and allowing the encapsulated compounds to penetrate deeper layers of the skin. Furthermore, the nanoscale dimensions of the particles allow for greater adhesion and surface contact area, which, in turn, improves the permeation of the compound through the skin [17]. They are, therefore, considered to be soluble substances [65]. In addition, it has been documented that the use of SLNs and NLCs in topical cosmetics reduces the likelihood that these carriers will cause systemic toxicity. Considering the points outlined above, SLNs and NLCs in cosmetic products are fully compliant with the relevant legislation and do not raise any safety concerns or objections. The current regulatory framework supports their use, confirming that they are safe and appropriate for inclusion in cosmetic formulations [34].

### 3.2. Food Supplements

The EC Regulation 2015/2283 defines engineered nanomaterials in food as novel foods, categorizing them as intentionally produced materials with dimensions of approximately 100 nm or less [66]. These materials can have discrete functional parts at the surface or internally, as well as structures, aggregates, or agglomerates, which may maintain nanoscale-specific properties despite having a size above 100 nm. Such properties may be related to specific physicochemical properties that differ from those of the non-nano form of the same material and/or to the large specific surface area of the materials considered. Nevertheless, the EU legislation applicable to novel food is the same as all food (Regulation EC 178/2002) [66]. Concerning this legislation, certain safety stipulations give rise to uncertainty, particularly the potential adverse effects of novel food on the health of both the current and future generations [67]. Regarding long-term effects, there is a gap in data to substantiate the safety of nanoparticles. However, the European Food Safety Authority (EFSA) has issued an exceptional document that provides an overview of the risk assessment of nanomaterials in food and the requisite information [68]. The document elucidates that lipid nanoparticles can facilitate the delivery of select food components, particularly those exhibiting pronounced lipophilicity. Moreover, the components of the nanoparticle can be derived from naturally occurring body lipids or approved food additives, such as emulsifiers. The primary safety concerns pertain to the extent of degradation of the encapsulation materials within the gastrointestinal tract. This encompasses not only the active ingredient itself but also the encapsulating material and the entire encapsulate/nanocarrier. In this regard, it has been proposed that specific adaptations in hazard characterization are required, including the assessment of the quantity of the encapsulated compound and the amount present in its free form in the food. Moreover, it may be advisable to examine the pertinent chemical constituents of a nanocarrier system and present evidence on how intestinal cells absorb and transport them [69].

In the USA, the FDA adheres to the same position as the EFSA when monitoring the safety and effectiveness of nanotechnology products. As well as regulating these products within its current regulatory framework, they are customized to the specific standards for each product category under its authority. Thus, the industry is committed to ensuring compliance with all applicable legal requirements and is encouraged to collaborate with the FDA to address any concerns regarding the safety or regulatory status of these products [64].

It is noteworthy that the safety of SLNs and NLCs in food supplements has been reported. Once ingested, these nanoparticles undergo the same physiological processes as the lipids present in food. This process includes digestion in the stomach, absorption primarily in the small intestine, and systemic blood uptake. In the small intestine, SLNs and NLCs can be broken down by lipase enzymes, releasing and facilitating the absorption of the compounds. Additionally, lipid nanoparticles have adhesive properties, allowing them to adhere to the gut wall, specifically the enterocyte surface, leading to the prompt absorption of the compounds within the enterocytes. Additionally, oral SLNs and NLCs are generally larger than 100 nm and biodegradable, indicating that their cellular uptake is not expected. Therefore, it is reasonable to conclude that no significant toxicological concerns are expected [70,71].

## 4. Future Perspectives

Most current research on encapsulating bioactive marine compounds in SLNs and NLCs is limited to in vitro studies, with minimal guidance on regulatory and safety standards for human application. To advance this field, future research should prioritize standardizing clinical trial protocols to facilitate comparisons across studies from different research groups; performing in vivo studies to better understand the absorption, distribution, and excretion of bioactive compounds and their respective nanocarriers; extending testing to human volunteers to confirm that findings from animal studies are applicable to humans; and publishing “negative results” to reduce redundancy, foster transparency, and accelerate collective learning.

Collaboration between academia and industry is also crucial to overcoming technological and financial challenges. Such partnerships drive innovation, bridge the gap between theory and practice, and help align research with real-world needs. Additionally, keeping regulatory standards in sync with the latest scientific developments is essential to ensure both safety and progress.

## 5. Conclusions

In recent years, the growing focus on sustainability and well-being has driven research into bioactive compounds from natural sources that are safe for human consumption. Marine bioactive compounds, in particular, have garnered attention for their potential health benefits. Despite their promise, these molecules often face challenges, such as poor stability and low water solubility. To address these issues, lipid nanoparticles, like SLNs and NLCs, have proven to be effective delivery systems, enhancing the stability and bioavailability of these compounds, protecting them from degradation, and improving their solubility in aqueous environments. This makes them more versatile for applications in healthcare, particularly in cosmetics and food supplements. In addition, SLNs and NLCs are often considered safer for human consumption and topical use compared to polymeric delivery systems, as lipids are naturally occurring substances.

Compared to other nanocarriers, such as liposomes, nanoemulsions, and polymeric nanoparticles, SLNs and NLCs offer several advantages, such as enhanced stability, lower toxicity, and simpler formulation processes. For instance, liposomes, while also effective at encapsulating bioactive compounds, can be more expensive and less stable in certain formulations. Nanoemulsions, though offering improved solubility, may have issues with stability. Polymeric nanoparticles can offer targeted delivery, but they are often associated with more complex production processes and potential biocompatibility concerns.

Despite their promising advantages, SLNs and NLCs have limitations that need to be addressed, such as the development of more comprehensive regulatory standards to ensure their safe use in healthcare products, particularly in cosmetics and food supplements. Additionally, further clinical and pre-clinical studies are required to establish their full safety profile, especially concerning long-term use in humans.

In conclusion, while SLNs and NLCs present an exciting solution for improving the delivery of bioactive marine compounds, more research, particularly in vivo studies, is essential to confirm their efficacy, safety, and broader applicability in healthcare products.

## Figures and Tables

**Table 1:** Relevant studies and applications of the main bioactive marine compounds encapsulated in SLNs and NLCs.

Marine Bioactive Compound	Type of Lipid Nanoparticle	Type of Study	Relevant Results	Healthcare Application	References
Docosahexaenoic and α-linolenic acid	SLN	In vitro	⇒ Encapsulation in SLNs improved the docosahexaenoic acid (277.2%) and α-linolenic acid (222.7%) uptake by colorectal cancer cells. ⇒ Docosahexaenoic acid-loaded SLNs showed greater inhibition of cell growth (68.6% and 80% in colorectal adenocarcinoma—HT29 and human colon cancer cells—and HCT116 cells, respectively) when compared to free docosahexaenoic acid at the same concentration (29.7% and 55.3% in HT-29 and HCT116 cells, respectively). ⇒ In HT-29 cells, α-linolenic acid-loaded SLNs were significantly more effective (*p* < 0.02) in inhibiting tumor cell growth than free α-linolenic acid at all the concentrations tested (5 µM, 10.7% vs. 34.6%; 10 µM, 12% vs. 36.3%; 50 µM, 2.3% vs. 38.2%). ⇒ In HCT116 cells, α-linolenic acid-loaded SLNs were significantly more effective in inhibiting tumor cell growth than free α-linolenic acid at concentrations of 10 and 50 µM (22.5% vs. 29%, *p* < 0.05 and 29% vs. 79.1%, *p* < 0.001, respectively).	Food supplement	[36]
Docosahexaenoic acid	NLC	In vitro and in vivo	⇒ NLC-loaded docosahexaenoic acid showed improved anti-inflammatory and antioxidant properties when compared to free docosahexaenoic acid, with DPPH of 0.57 ± 0.03 and 0.17 ± 0.003, respectively. ⇒ In vivo studies show that NLC-loaded docosahexaenoic acid had the most effective suppression of gingival inflammation compared to the pure free compound.	Food supplement	[37]
β–carotene	NLC	In vivo	⇒ In vivo permeation studies in humans show that β-carotene-loaded NLC improved skin penetration when compared to pure β-carotene: 34% of the encapsulated compound reached the deeper layers of the skin, while 45% of pure β-carotene was retained in the outer layers of the stratum corneum.	Cosmetic	[38]
		In vitro	⇒ β-carotene-loaded NLC demonstrated higher bioavailability (60.7%) and antioxidant properties (ABTS: 91.47 ± 1.9%, DPPH: 24.72 ± 0.38%, and IC_50_: 7.0 μg/mL) when compared to a β-carotene emulsion, a β-carotene–Tween^®^ 80 phosphate-buffered solution, and a β-carotene–phosphate-buffered solution.	Food supplement	[39]
Astaxanthin	NLC	In vitro and ex vivo	⇒ Astaxanthin-loaded NLC demonstrated improved stability, sustained release (83.0 ± 3.4% at 48 h), skin permeability (174.10 ± 4.38 μg/cm^2^), and skin retention (18.60 ± 1.62 μg/cm^2^) properties when compared to pure astaxanthin.	Cosmetic	[40]
		In vitro and in vivo	⇒ In vitro studies in human fibroblasts show an ROS reduction of 81.6%, DNA damage reduction of 41.6%, and cell death reduction of 62.69%. ⇒ In vivo studies show protection of all tested mice from skin damage.	Cosmetic	[41]
		In vitro	⇒ The antioxidant activity of astaxanthin was maintained after encapsulation in NLC: the results of DPPH were 95% for the free astaxanthin and 100% for astaxanthin-loaded NLC (*p* ≤ 0.05). ⇒ In vitro release studies show that compared to the free astaxanthin (90% in 12 h), the formulation exhibited a more sustained release profile (88% in 24 h).	Food supplement	[42]
	SLN and NLC	In vitro	⇒ Both astaxanthin-loaded NLC and astaxanthin-loaded SLNs were safe for nasal (RPMI 2650) and neuronal cells (SH-SY5Y) at concentrations up to 100 µg/mL, showing ability to inhibit neurodegenerative pathways, particularly oxidative stress.	Food supplement	[43]
	NLC	In vitro and in vivo	⇒ In vitro studies show a significant decrease in oxidative stress, neuroinflammation, amyloidogenic pathway, and apoptosis with astaxanthin-loaded NLC when compared to a free astaxanthin solution. ⇒ In vivo studies in mice show that astaxanthin-loaded NLC improved cholinergic neurotransmission when compared to a free astaxanthin solution.		[44]
Lycopene	NLC	In vitro	⇒ Lycopene-loaded NLC exhibited higher antioxidant capacity (36.6 ± 0.4 mM/mg and IC_50_: 14.1 ± 0.6mg/mL) when compared to placebo NLC (26.6 ± 0.1 mM/mg and IC_50_: 17.7 ± 0.4mg/mL).	Cosmetic	[45]
Fucoxanthin	NLC	In vitro	⇒ Fucoxanthin-loaded NLC was safe for concentrations up to 5 mM in human fibroblasts and significantly decreased the expression of psoriatic markers by approximately 40%.	Cosmetic	[46]
	SLN		⇒ The sun protection factor of fucoxanthin-loaded SLNs was significantly higher (1.85 times) than that of the other formulations tested (3 different chemical sunscreens).		[47]
Vitamin A	SLN	In vitro and ex vivo	⇒ 3400 ng of vitamin A was detected in the stratum corneum and upper epidermis when using vitamin A-loaded SLN formulation. After 24 h, the concentration increased in the deeper skin layers (from 0 to 250 ng).	Cosmetic	[48]
		In vitro, ex vivo and in vivo	⇒ In vitro studies show 54.38% of vitamin A release from SLNs in 24 h. ⇒ Ex vivo studies show that the concentration of vitamin A in the skin after applying vitamin A-loaded SLNs was almost 2 times higher when compared to a vitamin A hydrogel. ⇒ In vivo studies in mice showed an increased thickness of the stratum corneum and a primary irritation index of 0.00, after applying vitamin A-loaded SLNs.		[49]
	SLN and NLC	In vitro	⇒ Both vitamin A-loaded SLNs and vitamin A-loaded NLC demonstrated excellent stability under food processing conditions and gastric conditions, and it was observed that approximately 80% of the encapsulated vitamin A remained intact.	Food supplement	[50]
Vitamin D	NLC	In vitro	⇒ Vitamin D3-loaded NLC was more effective in inhibiting breast cancer cell proliferation (37 ± 5.1%) and increasing the percentage of cells in the apoptotic phase (40 ± 3.45%) when compared to a free vitamin D3 solution.	Food supplement	[51]
		In vivo	⇒ In vivo studies in mice show that the colon concentration of vitamin D3 was three times higher than the basal level and persisted for 12 h after the administration of vitamin D3-loaded NLC. Progressive mitigation of the symptoms was observed from day 3.		[52]
Vitamin E	NLC	In vitro	⇒ Vitamin E-loaded NLC was biocompatible with human skin keratinocytes (HaCAT cells) up to concentration of 10 μg/mL and showed an irritation score of 0.00.	Cosmetic	[53]
		In vitro and in vivo	⇒ In vitro studies show that vitamin E-loaded NLC demonstrated superior occlusive properties (80%) when compared to a vitamin E nanoemulsion. ⇒ In vivo studies in humans show that vitamin E-loaded NLC (12 g/H·m^2^) reduced the TEWL when compared to a vitamin E nanoemulsion (14 g/h·m^2^).		[54]
			⇒ In vivo studies in humans show no signs of erythema after 48 h of applying the vitamin E-loaded NLC. In addition, skin moisture increased by 80%, and skin elasticity improved significantly (*p* = 0.0319) over 12 weeks.		[55]
	SLN	In vitro	⇒ Hemolysis was minimal at all tested concentrations of vitamin E-loaded SLNs. ⇒ Vitamin E-loaded SLNs significantly boosted lymphocyte proliferation by approximately 150% and reduced DNA damage.	Food supplement	[56]
Vitamin K	SLN	In vitro	⇒ Vitamin K1-loaded SLNs showed stability in simulated gastrointestinal conditions and during storage.	Food supplement	[57]
Chitosan	NLC surface coating	In vitro and ex vivo	⇒ Chitosan-coated NLC showed excellent retention in the skin (581 ng after 4 h), biocompatibility, and efficacy in photodynamic therapy (50% reduction in melanoma cell viability after irradiation).	Cosmetic	[58]
Alginate	NLC surface coating	In vitro and ex vivo	⇒ Ex vivo studies in pig skin show that alginate-coated NLC originated high skin penetration of vitamin E (1.9-fold) and quercetin (2.3-fold) in damaged skin. ⇒ TEWL was reduced to −7 g/h·m^2^ when compared to the water control (3 g/h·m^2^).	Cosmetic	[59]
Gelatin	NLC surface coating	In vitro	⇒ Gelatin-coated NLC showed improved antioxidant and antimicrobial activities (0.1 ± 0.00 mg/mL) of the encapsulated compound for 90 days.	Food supplement	[60]

Abbreviations: ABTS: 2,2′-azino-bis(3-ethylbenzothiazoline-6-sulfonic acid) assay; DPPH: 2,2-diphenyl-1-pyridylohydrazinyl assay; NLCs: nanostructured lipid carriers; SLNs: solid lipid nanoparticles. TEWL: transepidermal water loss.
